# Supplementary material for: Are carbon emissions trading and green financial instruments synergistic? -Comprehensive quantitative research based on content analysis
Source: PLoS One. 2024 Mar 7;19(3):e0298601. doi: 10.1371/journal.pone.0298601 (PMC10919601; doi:10.1371/journal.pone.0298601)
Supplement: S1 Data — (ZIP) [file pone.0298601.s001.zip › Data/地级市Z_F工作报告.zip!/╕╩╦α/╛╞╚¬╩╨/└┤╫╘╣1⁄2╓┌║┼í╛┬φ┐╦╩2╛▌═°í┐_2013.docx]

各位代表：

　　现在，我代表酒泉市人民政府向大会作工作报告，请予审议，并请各位政协委员和其他列席人员提出意见。

　　2012年工作回顾

　　2012年，面对复杂严峻的国内外经济形势，全市各级深入贯彻省第十二次党代会、市第三次党代会精神和市三届人大一次会议精神，认真落实省委、省政府和市委决策部署，狠抓做精一产、做强二产、做大三产、做美城市、做实民生各项工作，全面开展“五个年”活动，攻坚克难，勇于担当，圆满完成了年初确定的目标任务。

　　——经济保持快速增长。预计全市生产总值576亿元，增长17%；财政总收入突破70亿元，财政总支出突破100亿元，分别增长18.2%和23.5%；公共财政收支分别增长24.9%和20.9%，均超额完成年度目标。

　　——转型跨越步伐加快。特色精品高效设施农业面积扩大、产出增加，占到农业总产值的65%；新能源、装备制造、现代高载能等主导产业规模扩大、比重提高，占到工业增加值的28%；物流、金融等现代服务业快速发展，占第三产业的比重提高1.3个百分点。

　　——投资消费增势强劲。开工建设重大项目579个，其中亿元以上项目259个，比上年增加143 个；预计限额以上固定资产投资完成800亿元，增长48%，年初确定的全社会固定资产投资“保900亿、争1000亿”的目标基本实现。社会消费品零售总额125.2亿元，增长19%。

　　——招商引资成效显著。落地建设招商引资项目443个，到位资金620亿元，增长1.56倍，引资总量及增速居全省第一。实现外贸进出口总额6934万美元，增长20%。引进利用外资2560万美元，增长4.6倍。

　　——人民生活更加殷实。城镇登记失业率控制在2.98%，居民消费价格涨幅控制在3%。预计城镇居民人均可支配收入突破2万元、农民人均纯收入达到9450元，分别增长16.2%和15.8%，移民人均纯收入实现20%的增长目标。政府承诺的高校毕业生就业、校安工程、保障房建设、集中供热等10件惠民实事全面落实，各项约束性指标全面完成。

　　这些成就的取得，标志着我们在科学发展的伟大实践中又迈出了坚实的一步。一年来的主要工作是：

　　1、坚持做精一产，大力发展现代农业。以服务城市、富裕农民为目标，大力发展现代设施农业、循环农业和高效节水农业，农业生产水平显著提高，预计第一产业增加值65亿元，同比增长6%。加大强农惠农力度，各级财政投入“三农”资金17.8亿元，增长19.8%，市财政设立扶持资金4700万元，支持发展特色优势产业、设施高效农业、精品珍品农业。启动实施肃州国家级现代农业示范区和敦煌省级现代农业示范区建设，新增现代制种、优质林果、日光温室、中药材等特色产业27.5万亩，全市3000元以上高效田达到125万亩。狠抓设施规模养殖，新续扩建养殖小区150个，规模养殖场户达到2.5万户，新增肉牛1.1万头、肉羊30万只，牛羊饲养量达到646万头（只）。积极推进农业产业化经营，扶持发展特色专业村126个，建成市级以上农民专业合作示范社150个，新建农业产业化项目43个，年销售收入5000万元以上的农业产业化龙头企业达到30户，农产品加工增值率达到53.4%。大力发展精耕细作农业，推进标准化种养技术和无公害农产品认定，加强农产品质量检测和畜禽疫病防控，农产品质量安全率达到99%。组织农业科技培训21.45万人次，完成技能培训3.75万人，输转农村劳动力16.9万人，实现劳务收入19.3亿元，增长18%。坚持实施封滩育林、禁牧休牧、“三北”五期防护林工程，认真落实耕地保护和草原生态保护奖补政策，完成造林17.3万亩，义务植树372万株，草原围栏面积达到360万亩，基本草原划定通过省上验收。敦煌水资源合理利用与生态保护综合规划启动实施，“引哈济党”工程通过国家水利部审查，北大河生态补水工程建成蓄水，新建水利工程108项，推广农田节水84万亩。深入开展“联村联户、为民富民”行动，制定实施新一轮移民集中安置区扶贫开发规划，落实扶贫资金2.3亿元，发展特色种植15万亩，肉羊饲养量突破30万只,比上年增加5万只，完成11个移民村整村推进建设任务，移民区生产生活条件明显改善。

　　2、着力做强二产，推动工业加速转型。加大工业支持力度，市级财政设立工业发展专项资金3000万元，扶持做强主导产业，改造提升传统产业，整合重组中小微型企业上规模，工业转型发展步伐加快，预计全市工业增加值达到257亿元，增长22.5%。加快新能源基地建设，风电二期首批300万千瓦项目核准开工建设，大型风机示范项目和光电建设项目进展顺利，全市建成和在建风电装机1020万千瓦、光电装机2019兆瓦。积极推进调峰电源建设，肃州电厂和瓜州常乐电厂项目前期工作完成，青羊沟等一批水电站建成发电，新增水电装机7万千瓦，累计达到39.9万千瓦。加快电网建设步伐，敦煌－鱼卡－格尔木750千伏输变电工程和一批配套电能外送与消纳的330千伏、110千伏输变电工程开工建设。装备制造产业链配套延伸，正泰150兆瓦太阳能电池组件生产线建成投产，京城控股电机制造、万盛太阳能等项目开工建设，风机设备年生产能力达到400万千瓦，光伏组件产能达到200兆瓦。大力推进资源勘探开发，三新硅业一期工程建成投产，并与国土资源厅、三新硅业签署三方战略合作协议，一批煤化工、水泥、矿产冶炼等产业链项目开工建设。主导产业引领工业转型，特别是电力、冶金、建材、石化、装备制造等产业规模扩大，配套产业链加速形成，实现增加值176亿元，对工业增长的贡献率达到90%。完善工业园区基础设施，3个市级园区和5个县级工业集聚区入驻企业689户，吸纳就业5.1万人，培育发展规模以上工业企业14户，新增年销售收入亿元以上企业8户，园区工业增加值达到216亿元，占工业经济总量的84%，园区聚集功能显著增强。

　　3、全力做大三产，深化服务业综合改革。强化第三产业发展措施，设立服务业财政引导资金6000万元，用于支持服务业综合改革，服务业与工业竞相发展的格局基本形成。预计，第三产业增加值超过200亿元，增长15%。繁荣活跃商贸流通，一些重点商业广场开工建设，一批大型商贸集散中心建成，精品商业街、特色美食街建设项目启动实施，新建便民肉菜店和农产品直销店50个，改造提升农家店100个，城乡市场规模扩大，酒泉被确定为全国电子商务示范基地。引强做大物流产业，举办2012中国物流万里行暨酒泉现代物流招商会，酒泉亚飞、瓜州柳沟等现代物流园建成，酒嘉综合物流园、巨龙农网物流园等物流项目开工建设，我市被评为全国新能源物流先锋城市。促进文化旅游融合发展，推进华夏文明保护传承和创新发展示范区建设，建成酒文化博览园等15个文化产业项目，鸣沙山·月牙泉5A级景区创建完成基础设施建设，举办飞天艺术旅游节、金秋胡杨节等旅游促销活动，全市接待游客802万人次，增长31%；实现旅游收入68.7亿元，增长32%。积极发展生产性服务业，建成省级以上工程技术研究中心和重点实验室7个、市级工程技术研究中心23个。推进金融体系建设，与中行甘肃分行等9家金融机构签署战略合作协议，甘肃银行酒泉办事处和酒泉农村商业银行成立，新增小额贷款公司和融资担保公司14家。深化银政银企合作，举办第九届银政银企洽谈会，签约贷款金额1319亿元，投放中小企业贷款179.9亿元，引入信托投资2.5亿元，贷款余额达到366.7亿元，增长15.5%。强力推进非公经济发展，全市新增私营企业994户、个体工商户7446户，累计达到5770户和4.1万户，非公经济增加值占经济总量的47%，比上年提高2.5个百分点，吸纳就业突破10万人，占非农从业人员的33%，缴纳税金24亿元，对地方财政的贡献达25%。

　　4、立足做美城市，着力改善城乡面貌。以建设国际明星城和戈壁明珠城为目标，坚持“以人为本、以水为脉、以绿为基”，着力打造体现文化内涵和地域特色魅力城市。高起点规划城市，酒嘉一体化城市总体规划纲要和酒泉市城市总体规划批准实施，新城区二期、北大河景观带等10个控制性详规和专项规划编制完成，瓜州城市总体规划批复实施，城乡规划体系实现全覆盖。高标准建设城市，酒泉城区重点实施城市博物馆等27项市政工程，全市完成城市建设投资46.3亿元，增长1.48倍。酒嘉城际一级公路提前建成通车，新建改造北滨河路等6条市政道路，热电联供面积达到400万平方米，新增天然气用户1.96万户，累计达到2.5万户，园林绿化、市容美化、城乡结合部综合整治卓有成效，人均公共绿地面积达到9平方米。县市城区建设步伐加快，金塔新城开发及城区环境治理、玉门新市区功能配套、瓜州城市绿化及景观建设、敦煌城区道路改造、肃北供水净化改造和阿克塞集中供热等市政项目建成，瓜州、肃北创建省级园林城市通过考核评审。高水平经营城市，完善土地收储机制，实现国土资源收益13.8亿元；采取财政担保信托等方式筹集资金7.8亿元，确保了重点市政工程的资金需求。积极推进城乡一体化，新建、改造农村公路700公里，肃阿公路、金嘉公路开工建设，瓜星高速公路建成通车，酒泉至航天城、柳园至敦煌高速公路列入国家和全省“十二五”规划；兰新铁路第二双线酒泉段进入铺轨施工，敦格铁路开展征地拆迁工作，酒泉公路客运枢纽站完成主体工程。加快农村小城镇建设，12个农村重点集镇道路及供排水设施全面建设，硬化村组街道267条，改造农村危旧房和残疾人危房1.58万户，建成楼居式中心村42个，新增农村安全饮水居民5.9万人。组织实施酒泉工业园区循环化改造项目，扶持13户企业开展资源回收循环利用改造，强化单位生产总值能耗和重点用能企业预警监测，节能降耗和污染减排实现省定目标。

　　5、聚焦做实民生，促进社会和谐进步。坚持民生优先，各级财政投入社会事业和民生资金43亿元，增长28%。深入开展“全民创业年”活动，扶持创业实体5839个，新增担保基金3169万元，发放创业贷款和妇女小额担保贷款18.2亿元，稳定和带动城乡就业9.4万人，我市被评为全国创业先进城市。实施积极的就业政策，落实就业补贴资金1.02亿元，招考公务员250名，招录444名高校毕业生到基层服务，选拨1027名高校毕业生到企业就业，新增城镇就业1.8万人。社会保障制度建设取得重大进展，推行城乡居民养老保险制度，参保率达96%。扩大城镇职工基本养老、医疗、失业、工伤、生育保险覆盖面，提高企业退休人员养老金、失业保险金和工伤人员保险待遇，城镇居民医疗保险和新农合补助标准提高20%，参保率均保持在98%以上。提高城乡低保、五保供养标准，完善医疗救助、临时救助等社会救助制度。保障性住房建设超额完成年度任务，新增保障性住房4690套，发放租赁补贴1.28万户。加强房地产市场调控，商品房价格涨幅控制在3%。开展科技特派员创业行动，示范推广新技术269项，“863”智能化种子装备技术研究项目启动实施，专利申请量突破700件，增长56%。扎实推进校舍安全工程和薄弱学校改造工程，消除危旧校舍10.2万平方米，新建校舍15.7万平方米，建成市级标准化学校20所，酒泉职业技术学院国家骨干高职院校项目通过评估验收，我市被评为全国“两基”工作先进市。加快实施学前教育三年行动计划，新续扩改农村幼儿园30所，城乡学前教育实现全覆盖。深化医药卫生体制改革，落实乡村医生补助政策，县级公立医院基本药物和医用耗材全部实现“零差率”,部分县级综合医院扩建、中医院建设和乡镇卫生院、村卫生室改造任务完成，95%的基层医疗卫生机构开展中医药诊疗服务。全面完成人口计生信息化建设，为22万名适龄妇女免费进行“两癌”普查和救助，孕前免费健康检查和幸福家庭创建活动扎实推进。继续深化文化管理体制改革，文化“三馆”和乡镇文化站全部免费开放，农家书屋实现村级覆盖，瓜州玄奘博物馆等一批公益文化设施开工建设，锁阳城、居延遗址等文化遗产保护项目顺利推进，举办广场文化艺术节等文化节会，广播电视村村通、户户通完成年度目标。广泛开展群众性体育活动，成功举办全国沙滩排球巡回赛、市第三届运动会等重大赛事。强化安全生产监管，狠抓重点行业和重点领域安全综合治理，安全生产形势总体稳定。深入开展食品药品专项整治，食品药品安全监管网络覆盖率达100%。加强“六五”普法宣传，深化矛盾纠纷大排查大调解，开展信访积案化解年、进京非正常上访治理等专项活动，一批信访积案和群体性上访事件得到解决。进一步完善综合治安防控体系，严厉打击各类违法犯罪活动，重大刑事案件下降37.7％，群众安全感明显增强。深化国防动员和双拥共建，我市被评为全国双拥模范城。依法加强宗教事务管理，开展民族团结进步创建活动，加大项目支持和对口帮扶力度，争取专项扶持资金1240万元，落实部门对口帮扶资金2293万元，“两个共同”示范县建设进展顺利。统计调查和服务水平进一步提高，防空体系建设获国家先进，防震减灾、气象、妇女、儿童、台侨、档案、史志、外事、边防等工作取得新的成绩。

　　各位代表，一年来，我们加强政府自身建设，深入开展效能风暴行动和“五个年”活动，切实推进作风转变，着力提高行政效能，为工作落实提供了有力保障；完善议事决策机制，健全听证公示、专家论证、责任追究等制度，提高了科学决策水平；自觉接受人大法律监督、工作监督和政协民主监督，办理人大代表意见建议和政协委员提案326件，办结率达60%；坚持依法行政，强化履职责任考核，增强了依法办事能力；深化审批制度改革，取消合并行政审批11项，下放行政许可13项，在全省率先建立行政服务电子监察系统，规范了行政行为；创新财政预算管理，加大资金争取和项目融资力度，财政保障能力明显提升；加强政府廉政建设，成立全省首家公共资源交易中心，落实项目代建、工程招投标、土地矿产出让、政府采购“监管办”分离等制度，加大矿产资源交易、工程建设招标等重点领域违纪违规行为的查处力度，对预算执行和23名领导干部进行审计，从源头上预防和治理腐败。

　　各位代表，回顾一年来的工作，在经济下行压力加大、不利因素交替叠加的背景下，我们科学判断形势，认真研究市情，趋利避害，抢抓机遇，攻坚克难，以项目建设提振经济增长，以招商引资助推转型跨越，以效能风暴力促任务落实，取得了科学发展的骄人业绩，向党的十八大献上了一份厚礼。发展令人振奋，成果来之不易，这是市委坚强领导的结果，是市人大、市政协和社会各界大力支持的结果，是全市各族人民团结一心、艰苦奋斗的结果，凝聚了各级政府及工作人员的心血和汗水。在此，我代表市政府，向各位人大代表、政协委员和广大干部群众，向各民主党派、工商联、人民团体、离退休老同志，向驻酒人民解放军、武警官兵和公安干警，向中央、省属驻酒单位以及所有关心、支持酒泉发展的各界人士，表示衷心的感谢和崇高的敬意！

　　在总结成绩的同时，我们也清醒地看到，我市经济社会发展中还存在一些困难和问题，主要是受项目审批放缓影响，风电二期没有完成进度目标；高载能产业发展滞后，电能就地消纳和外送能力不足；文化旅游资源开发利用不够，产业规模与经济发展水平和旅游龙头地位不相适应；扶贫开发任务繁重，大幅增加移民收入、同步实现全面小康目标任重道远。同时，政府工作效率与人民群众的愿望和要求还有差距，一些部门创新能力不强，机关工作人员的服务意识还需加强。对此，我们将在今后的工作中认真改进和解决。

　　2013年主要任务

　　2013年，是全面贯彻党的十八大精神的重要一年，也是深入实施“十二五”规划的关键一年，做好明年的政府工作，对于建设富裕文明和谐幸福酒泉、实现转型跨越发展具有十分重要的意义。

　　2013年政府工作的总体要求是：以党的十八大精神为指导，深入贯彻省第十二次党代会和市第三次党代会精神，以转型跨越为战略基点，大力实施“3341”项目工程，更加注重经济建设、生态建设、文化建设、社会建设和党的建设，全力打好做精一产、做强二产、做大三产、做美城市、做实民生五大攻坚战，继续深入开展“五个年”活动，力争在全省率先全面建成小康社会。

　　全市经济社会发展的预期目标是：地区生产总值增长16%，限额以上固定资产投资增长30%，财政收入增长16%，社会消费品零售总额增长18%，城镇居民人均可支配收入增长14%，农民人均纯收入增长12%，居民消费价格涨幅控制在4%以内，城镇登记失业率控制在4%以内，人口自然增长率控制在5.5‰以内，万元生产总值能耗降低3.2%，主要污染物排放完成约束性指标。为实现上述目标，我们将重点抓好八个方面的工作：

　　1、狠抓重大项目建设，培育壮大首位产业。坚持项目带动战略不动摇，以大项目拉动大投资、培植大财源、促进大发展。建立重大项目合力推进机制，实行百亿元产业链市级领导包抓责任制，对亿元以上重大项目成立协调推进小组，全面推行“一站式、直通车、全程化”服务，确保重大项目招得来、留得住、快发展。认真研究国家产业政策和投资导向，完善项目谋划机制，实行储备动态管理，做强做大以新能源及装备制造为主的首位产业，超前谋划基础设施、生态环境、社会民生等方面的重大项目，争取更多的项目列入国家和省上“盘子”,努力形成实施一批、申报一批、储备一批的项目建设格局。狠抓财源项目建设，在全面提高财政收入水平的基础上，着力增强市级财政保障能力。充分发挥通道优势，加大交通基础设施建设力度，实施敦煌机场扩建工程，加快肃阿公路、瓜州至敦煌快速通道、兰新铁路第二双线和敦格铁路建设，做好酒航铁路、酒航高速公路、敦煌至当金山高速公路等项目前期工作。加强对外交流合作，落实领导干部和部门单位招商引资责任，着力提高招商项目的合同履约率、开工竣工率和资金到位率。

　　2、推进工业转型升级，提升工业发展水平。大力实施工业强市战略，同步推进调峰电源、输出电网和资源综合利用，加快形成清洁能源与优势资源互促共进的新型工业体系，力争工业增加值增长20%以上。加大新能源基地建设力度，完成风电二期首批300万千瓦项目，实现65万千瓦大型风机示范项目并网发电，争取风电二期后续500万千瓦项目早日核准，基本建成全国首个千万千瓦级风电基地；积极争取光电项目并网指标，推进光伏、光热发电和风光互补、风光储一体化项目建设，新增光电装机1000兆瓦，力争把酒泉列入全国100个新能源示范市和国家分布式光伏发电规模应用示范区。争取酒泉—株洲±800千伏直流输变电工程核批建设，提高能源外送能力。集聚发展新能源装备制造业，重点引进风机配套设备、主要零部件制造和光伏材料生产项目，鼓励新能源企业优先采用本地风光电设备，争取国家风电研究检测中心测试基地、国家太阳能光伏发电系统质量检测中心实验室落户酒泉。加快调峰电源建设，争取开工建设肃州电厂和瓜州常乐电厂，新增水电装机5万千瓦，促进多能互补发展，着力提高调峰能力。大力推进找矿突破三年行动，引导矿产资源向优势企业集中，促进矿产品粗加工向精深加工转变，提高矿产资源开发水平和增值效益。坚持以资源换装备、以资源兴产业，扶持发展冶金、石化、煤化工、硅材料等现代高载能产业，争取将我市列为国家新能源综合利用示范区，推进三峡循环经济产业园建设，加快玉门浩海煤化工、瓜州淡水河谷煤化工、源森钒钛合金等高载能项目建设进度，促进电能就地消纳。科学编制新能源示范城市及工业园区规划，积极创建酒泉国家级经济技术开发区，大力推进“一区多园”和“飞地建园”，增强工业园区创新发展能力。

　　3、发展壮大现代农业，力促农民稳步增收。坚持服务城市、富裕农民不动摇，大力发展富民多元产业，力促农业转型、农民增收。加大强农惠农富农政策支持力度，确保各项农牧业奖补资金及时足额发放。深入实施蔬菜、草食畜等产业发展规划，建成万亩蔬菜专业大乡10个、千亩连片钢架大棚示范村20个、百亩连片日光温室示范区20个，新增3000元以上高效田20万亩，万元田种植规模达到22万亩。继续实施牛羊大县建设，促进生态牧业大发展，新续扩建规模养殖小区100个，新增肉羊25万只，新建农村户用沼气900户。推进农业产业化经营，扶持壮大农产品精深加工和运销龙头企业，加快建设大型冷链设施，农产品加工增值率达到57%以上。加强农产品质量监测体系和动物卫生安全体系建设，普及推广标准化和无公害生产技术，农产品质量安全合格率稳定在99%以上。推进土地承包经营权流转，吸引资本、技术等要素向农业聚集，加快建设现代农业示范园区，新增土地流转面积4万亩。组织农业科技培训20万人次，输转劳动力16万人次，力争人均创收1.2万元以上。统筹推进“联村联户行动”和“扶贫攻坚行动”，全面实施新十年扶贫开发纲要，争取落实财政专项扶贫资金6000万元，实施整村推进项目10个，整治改良土地5万亩，发展特色种植3万亩，新增肉羊养殖5万只，减少贫困人口1.5万人，力争移民人均纯收入增长20%以上。

　　4、大力发展第三产业，培育新的经济增长极。深入开展服务业综合改革试点，落实促进现代物流、商贸流通、旅游文化产业发展的政策措施，探索建立扩大消费需求长效机制，促进服务业特别是现代服务业发展壮大，力争第三产业增加值增长14.5%。启动实施酒嘉城际公路两侧商贸综合体建设规划，利用三年左右时间将其打造成为彰显汉唐文化、带动消费增长的特色街区。布局建设组团式商业集聚区、精品商业街和特色美食街，加快东方明都商业广场、义乌商贸城建设进度，新建改造一批专业批发市场、县乡集贸市场和鲜活农产品储藏库，推动农超对接，减少流通环节，营造便捷、廉价、安全的购物环境。加快国家物流节点城市和区域性物流中心建设步伐，加大酒嘉综合物流园、巨龙农网物流园等物流园区建设力度，支持敦煌机场建设国际航空口岸，力争全市物流总额增长18%。积极创建华夏文明保护传承创新发展示范区，加快建设敦煌文化动漫产业基地，建成丝绸之路博览园等产业园区，提高文化产业的规模化、集约化和专业化水平，力争文化产业增加值增长18%以上。争取开展国家旅游业综合改革试点，推进建设敦煌国家级文化旅游示范园区，办好敦煌行·丝绸之路国际旅游节和飞天文化旅游节，力争鸣沙山·月牙泉5A级景区验收达标，抓好大漠风车、自驾车游、徒步戈壁等新业态开发，力争接待游客突破1000万人次，增长25%，实现旅游收入86亿元，增长21%。加快奥凯种机、巨龙供销、敦煌旅游上市融资步伐，争取招商银行和浙商银行在酒泉设立分支机构，发展壮大小额贷款公司，完善金融服务和融资担保体系。以家政服务、社区服务、养老托幼、医疗保健等为重点，建立公益性信息服务平台，强化社区生活服务功能。加快建设社区放心粮油、蔬菜便利店，引导发展电子商务、食品配送等现代经营模式，开展农产品质量追溯体系建设试点，促进消费结构提档升级。积极推进全民创业，落实发展非公经济各项措施，做强做大非公经济，着力提高非公经济比重，使其成为推动转型跨越、富民强市的重要力量。

　　5、加快推进城镇建设，增强综合承载能力。把加快城镇化建设作为推动转型跨越发展的重要内容，着力建设“宜居、宜业、宜商、宜游”的国际明星城市和戈壁明珠城市。坚持规划引领城市，注重用文化元素提升城市品位，加强城市定位与历史文脉、自然空间、单体建筑及色彩的规划衔接，完成酒嘉新区、莫高湖区域等片区控制性详规，高起点编制酒嘉城际公路片区建设性详规，完善城市供热、供排水等专项规划。全面建设酒泉新城区二期道路交通、水电气热、垃圾处理、第二污水处理厂等基础设施和学校、医院、文体场馆、肃州区行政中心等公共服务设施，建成城市博物馆等地标性建筑。加快老城改造，积极创建全国园林城市，实施世纪湖公园改造、南滨河公园建设和城乡结合部、城市出入口绿化工程，新增公共绿地180万平方米。加快县城建设，推进建设小而精、小而美的特色城市，形成优势互补、风格迥异的现代城镇体系。高效经营城市，按照基础先行、市场配置原则，完善土地开发出让机制，盘活利用城市无形资源，采取多种融资方式，筹集城市建设资金。科学管理城市，深化数字城管建设，继续开展市容市貌集中整治活动，提升城市管理水平。统筹城乡发展，推进城乡规划、产业布局、基础设施、公共服务一体化，加快新农村建设，重点抓好农村集镇建设和交通要道、旅游景区周边农宅建设，改造农村危旧房1万户，新建百户以上社区化中心村20个，新增城镇人口1.1万人，城镇化率提高1个百分点。

　　6、加强生态环境保护，推进生态文明建设。着力构建生态安全屏障，推进绿色发展、循环发展、低碳发展，促进戈壁荒滩等未利用土地开发利用。严格控制用水总量，落实差别水价制度，抓好国家级高效节水灌溉示范项目和以色列政府贷款农田水利项目，力争肃州区进入全国规模化节水灌溉增效示范县行列。全面实施敦煌水资源合理利用与生态保护规划，继续抓好疏勒河、党河、黑河流域及中小河流治理，力争开工建设“引哈济党”工程。加强草原生态保护，开展生态补偿试验区、荒漠化治理示范区和湿地补偿试点工作，加快推进集体林权综合配套改革，继续实施封滩育林、禁牧休牧、“三北”五期防护林工程，完成人工造林10万亩，义务植树330万株。全力推进生产领域、居民生活和公共机构节能工作，降低能源、土地和水的消耗强度。组织实施一批节能环保项目,推进废弃物综合利用，强化水源地、大气、土壤等污染防治，继续实施百村连片整治项目。加快建设工业园区治污设施，开工建设河西地区危险废物集中处置中心和肃北、阿克塞污水垃圾处理项目，支持重点耗能企业进行减排技术改造,确保实现节能减排目标。

　　7、统筹发展社会事业，提升公共服务水平。坚持社会事业与经济建设同步发展，加大财政资金投入，形成政府主导、覆盖城乡、可持续的基本公共服务体系。深化科技体制改革，加快区域创新体系建设，支持科技型小微企业发展，组织实施国家和省级科技项目100项，示范推广实用技术150项。深化教育领域综合改革，健全义务教育考评机制，大力推进教育公平，促进教育均衡发展。普及农村学前教育，加快发展民族教育，突出抓好校安工程和学校标准化建设，改善农村、农垦团场和移民区办学条件，扩大城区学校办学规模。优化整合职教资源，成立兰州理工大学新能源学院。充分发挥文化引领风尚、教育人民、服务社会、推动发展的作用，实施重点文化项目建设工程，推动公共文化服务设施免费开放，实现农家书屋和广播电视自然村全覆盖。加快构建全民健身服务体系，做好全市第七届少数民族运动会筹办工作，积极争取在乡镇、社区实施体育惠民工程，推进市体育馆、游泳馆建设项目。深化医疗卫生体制综合改革，完善疾病预防控制、卫生监督和妇幼保健体系，实施孕产妇免费健康检查、重大传染病、地方病预防等公共卫生服务项目，建立重特大疾病保障和救助机制，扶持发展中医药事业，努力为群众提供安全、便捷的基本医疗服务。深化人口计生综合改革，落实计划生育利益导向政策。加强民族团结和宗教事务管理，深入推进“两个共同”示范县建设，促进民族共同繁荣发展。大力开展法制宣传教育，加强法律服务、法律援助和社区矫正工作，积极开展双拥创建活动，加强残疾人保障和服务体系建设，推进防震减灾项目建设，重视青少年教育成长，发展妇女儿童事业，认真做好统计、供销、台侨、档案、史志、外事及边防工作。

　　8、加大民生改善力度，增进人民群众福祉。实施就业优先战略和更加积极的就业政策，深入推进全民创业行动，健全政策扶持、创业服务、创业培训“三位一体”的创业带动就业机制，推动各类群体实现更高质量的就业，巩固创业型城市创建成果。全面建设覆盖城乡的社会保障体系，研究制定城镇企业职工、城乡居民、被征地农民和村干部养老保险转移接续办法，引导下岗失业人员、非公经济从业人员积极参加基本养老保险，探索建立城镇居民基本医疗保险门诊统筹制度，鼓励事业单位、社会团体、非公经济从业人员参加失业保险，推进机关事业单位参加工伤保险制度。完善社会养老服务体系，启动建设农村互助老人幸福院，提高居家养老服务覆盖面。加强社会救助管理，建立高龄老人补贴和居家养老服务补贴制度，及时发放“三老一少”和孤儿生活补助。强化价格监管，保持物价基本稳定。加强食品药品安全监管，确保饮食用药安全。落实安全生产责任，突出重点行业、重点领域执法监管，严防重特大事故发生。强化信访工作责任制和领导包案制，畅通群众诉求表达渠道，完善治安防控体系，扎实推进公安基础项目建设，提高人民群众的安全感和满意度。

　　切实解决民生问题，办好十件惠民实事：一是继续实施校舍安全工程，消除危旧校舍8万平方米，完成酒泉市六中、西大街小学改造搬迁工程；二是开工建设全科医生培训中心和市医院儿童专科、精神病专科；三是新建、改造农村道路600公里，再解决5万名农村居民的安全饮水问题；四是新增各类保障性住房4116套，发放租赁补贴1万户；五是全面启动实施北大河生态治理工程，确保完成年度目标任务；六是新增热电联供面积400万平方米，基本实现酒泉城区集中供热，天然气入户工程新增用户2万户；七是组织实施移民乡移民家庭劳动力技能培训和劳务输转全覆盖行动，力争两年内实现户均输转1名劳动力的目标；八是新建城市社区老年人日间照料中心8个、农村社区老年人日间照料中心25个；九是实施文化惠民工程，在城市社区和农村乡镇各建30个电子阅览室；十是继续推进“天眼”“百卡”工程，新建视频监控点1500个、智能卡口24个。

　　加强政府自身建设

　　党的十八大提出了建设中国特色社会主义的总依据、总布局和总任务，对新时期的政府工作提出了更高的要求。面对新的目标任务和人民群众的热切期待，我们必须更加注重政府自身建设，更加注重提高政府管理效能和服务水平，更加注重提升政府公信力和执行力，使政府工作符合转型跨越发展要求，努力建设人民群众满意的政府。

　　第一，坚持理论武装，不断提高驾驭全局能力。进一步加强各级政府党组织建设，大兴理论学习之风，把学习贯彻十八大精神作为当前和今后一个时期的首要政治任务，用党的最新理论成果武装头脑，统一思想，凝聚力量，正确把握形势，深化市情认识，运用战略思维，推动转型跨越，切实提高统揽全局、驾驭全局的能力和水平。

　　第二，坚持依法行政，不断提高科学施政能力。把依法行政贯穿到政府工作的各个领域、各个环节，进一步完善行政执法程序，严格按照法定权限和程序行使权力、履行职责，落实行政执法主体责任制和执法过错追究制。健全重大事项决策规则，完善公众参与、专家咨询、风险评估、合法性审查和集体讨论决定的决策机制。自觉接受人大法律监督、工作监督和政协民主监督，认真办理人大代表意见建议和政协委员提案。广泛听取民主党派、工商联和无党派人士的意见建议，虚心接受舆论监督和社会监督。

　　第三，坚持开拓创新，不断提高推动发展能力。制定完善规划，严格推进措施，加快建立全面建设小康社会落实机制。注重市场主体培育，强化生产要素保障，着力在项目谋划、招商引资等方面创新思路，善用重大政策做多项目数量，善用比较优势做大项目体量，善用产业转型做优项目质量，不断提高谋划项目、争取项目、落实项目的能力和水平。加快推进重点领域改革，着力构建保障发展、促进跨越的体制机制。

　　第四，坚持务实高效，不断提高执行落实能力。深入实施效能风暴行动，全面落实首问负责制、服务承诺制、限时办结制、责任追究制，建立健全工作协调机制，完善重大事项和重点工作跟踪落实机制，健全任务目标落实责任体系。深化审批制度改革，加强行政服务中心和公共资源交易平台建设，完善“一站式”服务。健全绩效考核评价和行政问责机制，科学合理确定评价标准，严格奖惩措施。深入开展调查研究，压缩精减会议文件，扎实推进工作落实。

　　第五，坚持廉洁从政，不断提高执政为民能力。认真落实廉政建设责任制，强化行政监察和审计监督，推进政府系统惩治和预防腐败体系建设。扩大政府信息公开，健全新闻发布制度，推进权力公开透明运行。加强对工程项目、财政资金使用、产权交易、土地出让、政府采购等重点领域和关键环节的监管，从源头上防治腐败。持续开展民主评议，坚决纠正行业不正之风。加强政府债务管理，建立预算绩效考评体系，严格控制“三公”经费。加强政府廉政建设，以廉政取信于民，以勤政造福于民，树立和维护政府良好形象。

　　各位代表，风劲潮涌，自当扬帆破浪；任重道远，更需快马加鞭。让我们在中共酒泉市委的坚强领导下，同心同德，砥砺前行，勇于担当，扎实工作，奋力开创建设富裕文明和谐幸福酒泉新局面！
